# Risk Factors of Early Liver Metastasis for Pancreatic Ductal Adenocarcinoma after Radical Resection

**DOI:** 10.1155/2022/8061879

**Published:** 2022-06-01

**Authors:** Jingshu Tong, Shengdong Wu, Changjiang Lu, Yong Yang, Shuqi Mao, Caide Lu

**Affiliations:** Department of Hepatopancreatobiliary Surgery, Ningbo Medical Centre Lihuili Hospital, Ningbo University, China

## Abstract

**Background:**

Liver metastasis arises in many postoperative patients with PDAC, occurring in the early stage appears to lead to a very poor prognosis.

**Objective:**

We aimed to analyze the risk factors for early liver metastasis after radical resection for patients with pancreatic ductal adenocarcinoma (PDAC) and to indicate the poor prognosis of early liver metastasis.

**Methods:**

Patients who underwent pancreatectomy for PDAC at the Ningbo Medical Centre Lihuili Hospital between January 2015 and June 2021 were included. The exclusion criteria were death within 30 days after the operation, complications with other malignancies, and a positive final resection margin (R1). Liver metastasis and its occurrence time were recorded, and risk factors for early (≤6 months) liver metastasis were analyzed by logistic regression models. The prognosis of patients with early liver metastasis and different recurrence patterns was analyzed by Kaplan–Meier curves and the log-rank test.

**Results:**

From the identified cohort of 184 patients, 172 patients were included for further analysis. 55 patients developed early liver metastasis within 6 months after the operation. Univariate analysis showed that CA125 ≥ 30 IU/ml, tumor size ≥ 4 cm, poor tumor differentiation, and portal vein/superior mesenteric vein (PV/SMV) reconstruction were risk factors, and multivariate analysis showed that poor tumor differentiation and PV/SMV reconstruction were independent risk factors for early liver metastasis. The prognosis of liver metastasis was the worst among the different recurrence patterns. Early liver metastasis indicates a poor prognosis in patients with PDAC.

**Conclusions:**

Poor differentiation and PV/SMV reconstruction are independent risk factors for early liver metastasis in patients with PDAC, and early liver metastasis indicates a poor prognosis.

## 1. Introduction

Globally, pancreatic ductal adenocarcinoma (PDAC) is the 12th most common malignancy and the 7th leading cause of cancer mortality [1]. Due to its extremely aggressive nature, radical resection is the only chance for long-term survival. However, even after curative radical resection, most patients will develop disease recurrence, resulting in a 5-year survival of only 12% to 27% [2, 3], and recurrence and metastasis negatively affect the curative nature of the operation and the prognosis of PDAC patients.

Liver metastasis has the worst prognosis among all the recurrence patterns in PDAC, and the median OS is only 15.4 months, while other recurrence patterns are 17.7-39.6 months [4]. Early recurrence is another indicator for poor prognosis, which may lead to a 21.6-month reduction in OS compared to late recurrence [5].

Early liver metastasis means liver metastasis within 6 months after operation [6], which may represent a unique biologic characteristic and always indicates a poor prognosis. Patients susceptible to early liver metastasis after surgery constitute a key cohort worthy of further study [5, 7]. In this study, we concentrated on “liver metastasis” in the “early stage,” which has not been reported in previous studies, to explore the risk factors for early liver metastasis, as these risk patients may benefit from a relevant adjuvant approach.

## 2. Patients and Methods

### 2.1. Patient Selection

**Figure 1:**
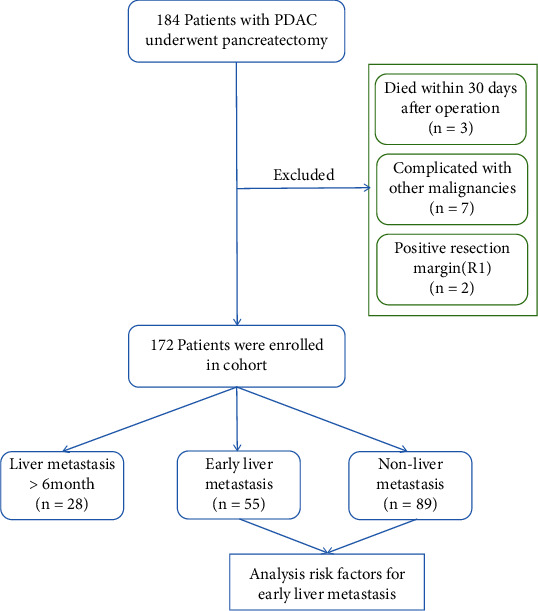
Exclusion criteria and grouping methods.

The study cohort consisted of 184 patients who underwent pancreatic cancer resection in the Department of Hepatopancreatobiliary Surgery, Ningbo Medical Centre Lihuili Hospital, between January 2015 and June 2021. According to the inclusion and exclusion criteria, 172 patients were enrolled. The inclusion criteria were as follows: (1) curative-intent pancreatectomy, (2) pathology confirmed PDAC, and (3) integrated clinical and follow-up data. The exclusion criteria were as follows: (1) death within 30 days after the operation, (2) complications with other malignancies, and (3) a positive final resection margin (R1) (Figure 1). The study was approved by the ethics committee of Ningbo Medical Center Lihuili Hospital (Approval number: KY2021PJ263).

### 2.2. Follow-Up

Patients were followed up until December 2021, and all 172 patients were followed up for more than 6 months unless they died. The median follow-up time was 15.5 months (3-69 months), and all enrolled patients were followed up for more than 6 months to ensure whether early liver metastasis occurred. In general, patients had at least 1 follow-up by imaging study (CT, MRI or PET/CT) and tumor biomarkers (CA199, CA125, and CEA) every 3 months for the first year after operation and then every 3-6 months after the first year. Follow-up was performed in the outpatient clinic or via phone call.

### 2.3. Early Liver Metastasis

The diagnosis of postoperative recurrence was based on imaging studies and rarely tissue confirmation. Early liver metastasis was defined as liver metastasis within 6 months after the operation [6]. In this study, we concentrated on early liver metastasis for those patients with single or multiple recurrences, and the time of liver metastasis was recorded.

### 2.4. Patterns of Recurrence

Recurrence patterns were determined by recording the initial site of recurrence and were stratified into four mutually exclusive categories: “liver metastasis,” “lymph node recurrence,” “disseminated recurrence,” and “multisite recurrence,” and multisite recurrence refers to multiple recurrence or metastasis at the time of initial diagnosis recurrence.

### 2.5. Operation and Definitions

Both CT and MRI were performed to evaluate resectability and exclude synchronous liver metastasis before operation. According to imaging studies and exploration during the operation, portal vein/superior mesenteric vein (PV/SMV) or celiac axis/common hepatic artery (CA/CHA) resection and reconstruction were performed if the tumor had invaded. Resection margins (*R*) of the pancreas, distal bile duct, PV/SMV, and retroperitoneal tissue were studied in detail for the microscopic presence of tumors. The absence or presence of tumor cells on the resection margins was used as a criterion for judging a negative (R0) or positive (R1) resection margin. A frozen section of the resection margin was usually performed, with additional resection if necessary, based on frozen resection analysis results. The lymph node ratio (LNR) was defined as the number of lymph nodes harboring cancer divided by the number of total nodes harvested. Disease stage was defined according to the 8th edition of the American Joint Committee on Cancer (AJCC) staging system [8].

### 2.6. Statistical Analysis

Quantitative variables are reported as the mean with standard deviation. Categorical variables are presented as absolute counts and percentages. Univariate and multivariate logistic regression models were used to analyze potential risk factors for predicting early liver metastasis. The prognosis of patients with early liver metastasis was analyzed by Kaplan–Meier curves and the log-rank test. The prognosis of different recurrence patterns was analyzed by Kaplan–Meier curves and the log-rank test. All factors with a *P* value of <0.05 in univariate analysis were included as covariate in multivariate regression analysis to identify independent factors. Difference was considered significant when *P* value <0.05. Statistical analysis was performed with SPSS 23.0 statistical software (SPSS Inc., Chicago, IL).

## 3. Results

### 3.1. Cohort Characteristics

Between January 2015 and June 2021, a total of 184 patients underwent pancreatectomy and had histologically confirmed PDAC at the Affiliated Lihuili Hospital of Ningbo University. Excluded from this cohort were 3 patients who died within 30 days after the operation, 7 patients with other malignancies, and 2 patients with a positive (R1) resection margin. After exclusion, all 172 patients with more than 6 months of follow-up were eligible for further analysis. The clinicopathological and treatment characteristics of these patients are summarized in Table 1.

### 3.2. Early Liver Metastasis and Its Recurrence Patterns

Among the 172 patients, 55 patients developed early liver metastasis within 6 months, 28 patients developed liver metastasis more than 6 months after the operation, and 89 patients without liver metastasis until the date of death or the last follow-up (Figure 1). The recurrence patterns of patients with early liver metastasis are summarized in Table 2.

### 3.3. Factors Associated with Early Liver Metastasis

Analysis of clinicopathological factors associated with early liver metastasis after operation, and univariate analysis showed that CA125 ≥ 30 IU/ml, tumor size ≥ 4 cm, poor tumor differentiation, and PV/SMV reconstruction were risk factors for early liver metastasis. Multivariate analysis showed that poor tumor differentiation and PV/SMV reconstruction were independent risk factors for early liver metastasis (Tables 3 and 4). Compared with liver metastasis after 6 months, CA125 ≥ 30 IU/ml was an independent risk factor for early liver metastasis (Table 5).

### 3.4. Survival of Patients with Early Liver Metastasis

**Figure 2:**
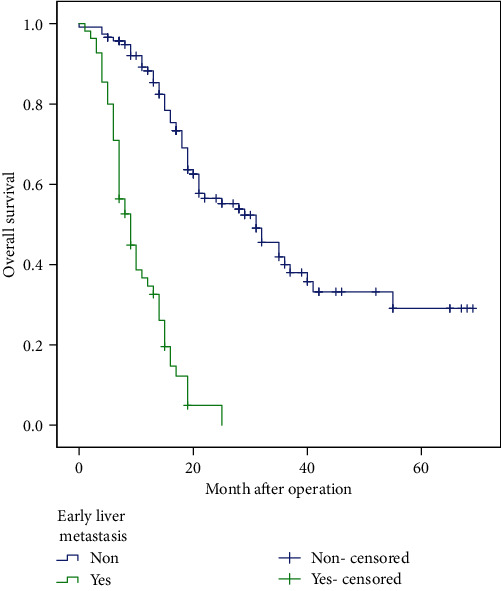
Overall survival curve for patients with early liver metastasis.

Early liver metastasis after operation for patients with PDAC indicates a poor prognosis. The median OS of all 172 patients was 19 months (95% CI 16.3-21.7), and the median OS of patients with early liver metastasis (*n* = 55, 9 months, 95% CI 6.7-11.3) was significantly shorter than that of patients without early liver metastasis (*n* = 117, 31 months, 95% CI 23.0-39.0) (*χ*^2^ = 89.37, *P* < 0.001, Figure 2).

### 3.5. Analysis Comparing Prognosis in Different Recurrence Patterns

**Figure 3:**
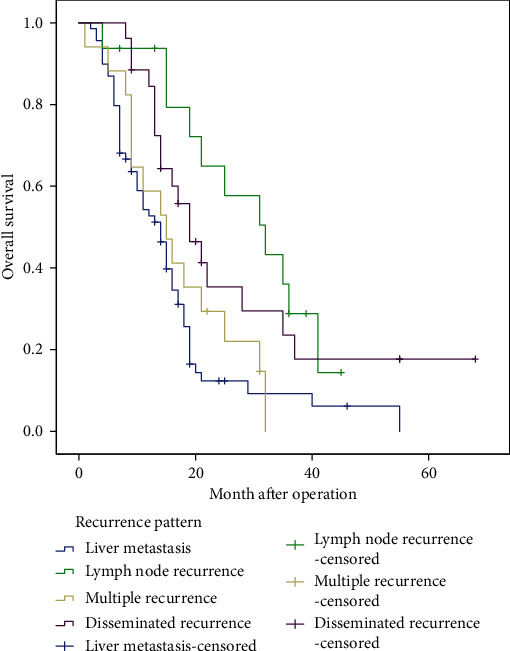
Overall survival curves for patients with different recurrence patterns.

In different patterns of recurrence after operation, patients with liver metastasis (*n* = 69, 14 months, 95% CI 10.9-17.1) had a significantly worse prognosis than those with lymph node recurrence (*n* = 16, 32 months, 95% CI 19.4-44.6, *P* = 0.001) or disseminated recurrence (*n* = 26, 19 months, 95% CI 13.4-24.6, *P* = 0.004). Patients with liver metastasis had a poor prognosis, which was not significantly different from multisite recurrence (*n* = 17, 15 months, 95% CI 8.3-21.7, *P* = 0.115) (Table 6, Figure 3).

## 4. Discussion

Patterns and stage of recurrence may lead to different outcomes in patients with PDAC, especially liver metastasis, which accounts for the largest proportion and poorest prognosis, resulting in an increase in mortality [9, 10]. Gastrointestinal system cancers tend to metastasize to the liver owing to venous blood returning through the portal vein circulation; as the “seed and soil” hypothesis implies, the reciprocal interactions between tumor cells and liver-recruited inflammatory immune cells have important roles in engraftment, tumor progression, and liver metastasis [11]. According to 27 patient autopsies, Hishinuma et al. [12] reported that local recurrence is rarely a direct cause of death, instead most patients died of liver metastasis.

The stage of recurrence is another key issue worth researching. Although the initial recurrence time is significantly related to the prognosis, there is presently no established or evidence-based definition for early recurrence of PDAC after pancreatectomy. Throughout the present literature, various cutoff values have been used to define early recurrence: 6 months by Sugiura et al. [13], Matsumoto et al. [14], and the National Comprehensive Cancer Network (NCCN) 2021 guidelines [6], 12 months by Vincent [5], and 8 months by Niedergethmann et al. [15]. In this study, the unique definition for early liver metastasis forms an innovation, and we believe 6 months as classifying early liver metastasis patients after operation, as liver metastasis will almost certainly arise within one year, and occurring in the early stage after operation appears to indicate a very poor prognosis.

This study demonstrates that poor differentiation and PV/SMV reconstruction are independent risk factors for early liver metastasis. We calculated that the probabilities of early liver metastasis were 43.8%, 23.8%, and 8.3% in patients with poor, moderate, and high tumor differentiation, respectively. The results are the same as those of a previous large sample prospective study [9], considering that poor differentiation associated with strong infiltration and invasion characteristics contributes to liver metastasis. Shibata et al. [16] offer an intriguing hypothesis, suggesting that epidermal growth factor receptor, E-cadherin, and laminin chain are expressed at high levels in poorly differentiated tumors, and may enhance the ability of PDAC to early metastasize to liver after pancreatectomy. Although poor differentiation has been recognized as an indicator of poor prognosis, the exact relationship remains unclear, and the biological mechanism remains to be further researched.

Another independent predictor for early liver recurrence is PV/SMV reconstruction. Due to the pancreas' adjacent anatomical relationship, the PV/SMV is a common site of tumor involvement by direct invasion. As a significant symbol of resectability of pancreatic cancer [6], extended pancreatectomy has been widely accepted and implemented in major centers, but there is still no consensus on survival or recurrence outcomes. A meta-analysis of nine studies reported statistically similar mortality and OS between extended pancreatectomy with PV/SMV resection versus standard pancreatectomy [17], but Addeo et al. [18] and Ravikumar et al. [19] reported that the depth of venous invasion is an independent risk factor for prognosis. In our study, tumor invaded PV/SMV reconstruction was a strong independent risk factor for early liver metastasis, and 41.1% of patients with PV/SMV reconstruction developed early liver metastasis. The “circulating tumor cell (CTC)” hypothesis may be used to explain this phenomenon: during extended pancreatectomy, the tumor cell invading the PV/SMV tends to fall off from the primary focus into blood circulation, through the PV and form liver metastasis. Tien et al. [20] supported this hypothesis by analyzing CTCs in PV blood obtained from patients with PDAC during operation, demonstrating that the detection of CTCs in the PV was associated with liver metastasis after operation. The above findings may help us to derive a deeper understanding of extended pancreatectomy.

This study found that overall survival after PDAC recurrence differed based on the patterns of recurrence. Patients with liver or multisite recurrence had a limited median overall survival of <15 months, while patients with lymph node recurrence had a median survival of >30 months, which was similar to a prior study, but they did not take “early-stage” into consideration [4]. Patients with early liver metastasis possibly represent another unique clinical and biological subtype of PDAC, in which more aggressive or localized additional therapy might be justified. Adjuvant chemotherapy has already proven to be an important measure to reduce recurrence and improve survival for PDAC [21], but in this study, adjuvant chemotherapy was not a protective factor for early liver metastasis, which may be caused by the mixture of other patterns of recurrence. However, timely adjuvant chemotherapy is still essential for patients with high-risk factors or who have already experienced early liver metastasis, and the relevant theoretical evidence will be further studied in our center. Although no standard adjuvant chemotherapy for PDAC has been established, several gemcitabine-based adjuvant therapies have been investigated [21], and previous studies reported that systemic intra-arterial chemotherapy appeared to be effective against liver metastasis [22, 23]. Furthermore, hepatectomy for recurrent PDAC was applied in a German national cancer center and was proven to be safe and beneficial [24, 25]. Now that both chemotherapy and hepatectomy have proven successful in improving survival, the relevant specific regimens are worth further study for patients with early liver metastasis after operation.

To analyze the risk factors for early liver metastasis, this study mainly compared the early liver metastasis group with the no liver metastasis group, which also helped to avoid some confounding factors. Several limitations in the study are worthy of mentioning. First, recurrence was generally based on radiographic findings without tissue confirmation, and tiny hepatic nodules are difficult to identify as recurrence or cyst, limiting the accuracy of the recurrence date. Second, there were no differences in postoperative adjuvant chemotherapy between early liver metastasis and nonliver metastasis in this study. The sampling error and selection bias caused by fewer cases in the early recurrence group may be the reason for this phenomenon.

## 5. Conclusions

This study reports that poor differentiation and PV/SMV reconstruction are the risk factors for early liver metastasis, and early liver metastasis indicates a poor prognosis. These findings are highly suggestive of biologic heterogeneity in PDAC patients with early liver metastasis. Future studies might reveal molecular genetic signatures associated with early liver metastasis, possibly exploiting prognostic stratification, targets of treatment, and a more patient-tailored approach for PDAC patients with early liver metastasis.

## Figures and Tables

**Table 1:** Clinicopathological and treatment characteristics of the 172 patients.

Variable	Total (*n* = 172)
Age (years, %)	
≥60	132 (76.7)
<60	40 (23.3)
Sex (%)	
Male	100 (58.1)
Female	72 (41.9)
BMI (kg/m^2^, %)	
≥24	55 (32.0)
<24	117 (68.0)
Tumor size (cm, %)	
>4	75 (43.6)
≤4	97 (56.4)
Tumor location (%)	
Head/neck	109 (63.4)
Body/tail	63 (36.6)
Lymph node metastasis (%)	
Yes	71 (41.3)
No	101 (58.7)
Lymph node ratio (%)	
≥0.2	32 (18.6)
<0.2	140 (81.4)
Capsule invasion (%)	
Present	58 (33.7)
Absent	114 (66.3)
Tumor differentiation (%)	
Poor	80 (46.5)
Well-moderate	92 (53.5)
Microvascular invasion (%)	
Present	87 (50.6)
Absent	85 (49.4)
Perineural invasion (%)	
Present	146 (84.9)
Absent	26 (15.1)
Frozen resection margin (%)	
Positive	20 (11.6)
Negative	152 (88.4)
PV/SMV resection and reconstruction (%)	
Yes	56 (32.6)
No	116 (67.4)
CA/CHA resection and reconstruction (%)	
Yes	4 (2.3)
No	168 (97.7)
Morbidity (%)	
Clavien–Dindo grades 0-II	165 (95.9)
Clavien–Dindo grades III-IV	7 (4.1)
Neoadjuvant chemotherapy (%)	
Yes	20 (11.6)
No	152 (88.4)
Adjuvant chemotherapy (%)	
Yes	113 (65.7)
No	59 (34.3)
TNM stage (%)	
I-IIA	101 (58.7)
IIB-IV	71 (41.3)

Data are presented as numbers (percentages). BMI: body mass index; PV/SMV: portal vein/superior mesenteric vein; CA/CHA: celiac axis/common hepatic artery.

**Table 2:** Recurrence patterns of patients with early liver metastasis (*n* = 55).

Early liver metastasis patterns	*n*
Liver metastasis only	49
Multiple	
Liver+retroperitoneum	2
Liver+locoregional	1
Liver+lung	1
Liver+retroperitoneum+lung	1
Liver+retroperitoneum+peritoneal+spleen	1

**Table 3:** Univariate analysis of risk factors for early liver metastasis.

Variable	Early liver metastasis (*n* = 55)	Nonliver metastasis (*n* = 89)	OR (95% CI)	*P*
Age (years, %)				
≥60	41 (74.5)	67 (75.3)	0.962 (0.443-2.086)	0.921
<60	14 (25.5)	22 (24.7)		
Sex (%)				
Male	37 (67.3)	52 (58.4)	1.463 (0.724-2.956)	0.289
Female	18 (32.7)	37 (41.6)		
BMI (kg/m^2^, %)				
≥24	15 (27.3)	30 (33.7)	0.738 (0.352-1.543)	0.419
<24	40 (72.7)	59 (66.3)		
ALB (g/L, %)				
≥40	23 (41.8)	42 (47.2)	0.804 (0.408-1.585)	0.529
<40	32 (58.2)	47 (52.8)		
ALT (U/L, %)				
≥40	25 (45.5)	35 (39.3)	1.286 (0.651-2.538)	0.469
<40	30 (54.5)	54 (60.7)		
CA199 (IU/ml, %)				
≥400	17 (30.9)	23 (25.8)	1.284 (0.611-2.699)	0.510
<400	38 (69.1)	66 (74.2)		
CA125 (IU/ml, %)				
≥30	17 (30.9)	14 (15.7)	2.397 (1.068-5.376)	0.034
<30	38 (69.1)	75 (84.3)		
CEA (ug/L, %)				
≥5	8 (14.5)	16 (18.0)	0.777 (0.308-1.957)	0.592
<5	47 (85.5)	73 (82.0)		
Tumor size (cm, %)				
>4	30 (54.5)	29 (32.6)	2.483 (1.243-4.957)	0.010
≤4	25 (45.5)	60 (67.4)		
Tumor location (%)				
Head/neck	39 (70.9)	50 (56.2)	1.901 (0.928-3.894)	0.079
Body/tail	16 (29.1)	39 (43.8)		
Lymph node metastasis (%)				
Yes	26 (47.3)	35 (39.3)	1.383 (0.701-2.728)	0.349
No	29 (52.7)	54 (60.7)		
Lymph node ratio (%)				
≥0.2	15 (27.3)	13 (14.6)	2.192 (0.951-5.056)	0.066
<0.2	40 (72.7)	76 (85.4)		
Capsule invasion (%)				
Present	19 (34.5)	27 (30.3)	1.212 (0.592-2.481)	0.599
Absent	36 (65.5)	62 (69.7)		
Tumor differentiation (%)				
Poor	35 (63.6)	34 (38.2)	2.831 (1.411-5.679)	0.003
Well-moderate	20 (36.4)	55 (61.8)		
Microvascular invasion (%)				
Present	33 (60.0)	40 (44.9)	1.837 (0.929-3.635)	0.080
Absent	22 (40.0)	49 (55.1)		
Perineural invasion (%)				
Present	47 (85.5)	74 (83.1)	1.191 (0.469-3.026)	0.714
Absent	8 (14.5)	15 (16.9)		
Frozen resection margin (%)				
Positive	4 (7.3)	10 (11.2)	0.620 (0.184-2.082)	0.439
Negative	51 (92.7)	79 (88.8)		
PV/SMV reconstruction (%)				
Yes	25 (45.5)	23 (25.8)	2.391 (1.173-4.874)	0.016
No	30 (54.5)	66 (74.2)		
CA/CHA reconstruction (%)				
Yes	2 (3.6)	1 (1.1)	3.321 (0.294-37.514)	0.332
No	53 (96.4)	88 (98.9)		
Morbidity (%)				
0-II	52 (94.5)	86 (96.6)	0.605 (0.118-3.108)	0.547
III-IV	3 (5.5)	3 (3.4)		
Neoadjuvant chemotherapy (%)				
Yes	6 (10.9)	11 (12.4)	0.868 (0.302-2.498)	0.793
No	49 (89.1)	78 (87.6)		
Adjuvant chemotherapy (%)				
Yes	32 (58.2)	61 (68.5)	0.639 (0.318-1.284)	0.208
No	23 (41.8)	28 (31.5)		
TNM stage (%)				
I-IIA	29 (52.7)	54 (60.7)	0.723 (0.367-1.426)	0.349
IIB-IV	26 (47.3)	35 (39.3)		

Data are presented as numbers (percentages). ALB: albumin; CA199: carbohydrate antigen 199; CA125: carbohydrate antigen 125; CEA: carcinoembryonic antigen. For abbreviations see Table 1.

**Table 4:** Multivariate analysis of risk factors for early liver metastasis.

Variable		*β*	Wald	OR	95% CI	*P*
CA125	≥30 IU/ml	0.577	1.632	1.781	0.735-4.315	0.201
Tumor size	≥4 cm	0.630	2.697	1.877	0.885-3.978	0.101
Tumor differentiation	Poor	1.129	8.699	3.093	1.461-6.550	0.003
PV/SMV reconstruction	Yes	0.903	5.059	2.467	1.123-5.417	0.024

For abbreviations, see Table 1.

**Table 5:** Analysis of risk factors by comparison with liver metastasis after 6months.

Variable	Liver metastasis		Univariate analysis		Multivariate analysis	
	Early (*n* = 55)	>6 mo (*n* = 28)	OR (95% CI)	*P*	OR (95% CI)	*P*
Age (years, %)						
≥60	41 (74.5)	24 (85.7)	0.488 (0.144-1.653)	0.249		
<60	14 (25.5)	4 (14.3)				
Sex (%)						
Male	37 (67.3)	11 (39.3)	3.177 (1.235-8.171)	0.016	2.755 (0.999-7.593)	0.050
Female	18 (32.7)	17 (60.7)				
BMI (kg/m^2^, %)						
≥24	15 (27.3)	10 (35.7)	0.675 (0.255-1.789)	0.429		
<24	40 (72.7)	18 (64.3)				
ALB (g/L, %)						
≥40	23 (41.8)	15 (53.6)	0.623 (0.249-1.557)	0.311		
<40	32 (58.2)	13 (46.4)				
ALT (U/L, %)						
≥40	25 (45.5)	11 (39.3)	1.288 (0.510-3.250)	1.288		
<40	30 (54.5)	17 (60.7)				
CA199 (IU/ml, %)						
≥400	17 (30.9)	6 (21.4)	1.640 (0.563-4.776)	0.364		
<400	38 (69.1)	22 (78.6)				
CA125 (IU/ml, %)						
≥30	17 (30.9)	2 (7.1)	5.816 (1.237-27.339)	0.026	5.872 (1.187-29.046)	0.030
<30	38 (69.1)	26 (92.9)				
CEA (ug/L, %)						
≥5	8 (14.5)	2 (7.1)	2.213 (0.437-11.202)	0.337		
<5	47 (85.5)	26 (92.9)				
Tumor size (cm, %)						
>4	30 (54.5)	16 (57.1)	0.900 (0.360-2.253)	0.822		
≤4	25 (45.5)	12 (42.9)				
Tumor location (%)						
Head/neck	39 (70.9)	20 (71.4)	0.975 (0.357-2.665)	0.961		
Body/tail	16 (29.1)	8 (28.6)				
Lymph node metastasis (%)						
Yes	26 (47.3)	10 (35.7)	1.614 (0.632-4.118)	0.317		
No	29 (52.7)	18 (64.3)				
Lymph node ratio (%)						
≥0.2	15 (27.3)	4 (14.3)	2.250 (0.669-7.572)	0.190		
<0.2	40 (72.7)	24 (85.7)				
Capsule invasion (%)						
Present	19 (34.5)	12 (42.9)	0.704 (0.277-1.788)	0.460		
Absent	36 (65.5)	16 (57.1)				
Tumor differentiation (%)						
Poor	35 (63.6)	11 (39.3)	2.705 (1.060-6.899)	0.037	2.123 (0.771-5.841)	0.145
Well-moderate	20 (36.4)	17 (60.7)				
Microvascular invasion (%)						
Present	33 (60.0)	14 (50.0)	1.500 (0.600-3.750)	0.386		
Absent	22 (40.0)	14 (50.0)				
Perineural invasion (%)						
Present	47 (85.5)	25 (89.3)	0.705 (0.172-2.896)	0.628		
Absent	8 (14.5)	3 (10.7)				
Frozen resection margin (%)						
Positive	4 (7.3)	6 (21.4)	0.288 (0.074-1.121)	0.073		
Negative	51 (92.7)	22 (78.6)				
PV/SMV reconstruction (%)						
Yes	25 (45.5)	8 (28.6)	2.083 (0.784-5.533)	0.141		
No	30 (54.5)	20 (71.4)				
CA/CHA reconstruction (%)						
Yes	2 (3.6)	1 (3.6)	1.019 (0.088-11.746)	0.988		
No	53 (96.4)	27 (96.4)				
Morbidity (%)						
0-II	52 (94.5)	27 (96.4)	1.558 (0.155-15.700)	0.707		
III-IV	3 (5.5)	1 (3.6)				
Neoadjuvant chemotherapy (%)						
Yes	6 (10.9)	3 (10.7)	1.020 (0.235-4.426)	0.978		
No	49 (89.1)	25 (89.3)				
Adjuvant chemotherapy (%)						
Yes	32 (58.2)	20 (71.4)	0.557 (0.209-1.482)	0.241		
No	23 (41.8)	8 (28.6)				
TNM stage (%)						
I-IIA	29 (52.7)	18 (64.3)	0.620 (0.243-1.581)	0.317		
IIB-IV	26 (47.3)	10 (35.7)				

Data are presented as numbers (percentages). For abbreviations, see Tables 1 and 3.

**Table 6:** Comparison of the prognosis of patients with different recurrence patterns.

Recurrence pattern	Liver metastasis	Lymph node recurrence	Disseminated recurrence	Multisite recurrence	Median OS months (95% CI)
Liver metastasis	NA	*P* = 0.001	*P* = 0.004	*P* = 0.401	14 (10.9-17.1)
Lymph node recurrence	/	NA	*P* = 0.326	*P* = 0.007	32 (19.4-44.6)
Disseminated recurrence	/	/	NA	*P* = 0.115	19 (13.4-24.6)
Multisite recurrence	/	/	/	NA	*n* (8.3-21.7)

## Data Availability

Data are available upon request through contacting authors by 1174608081@qq.com.
